# Supplementary material for: Study on the Suitability of Tea Cultivars for Processing Oolong Tea from the Perspective of Aroma Based on Olfactory Sensory, Electronic Nose, and GC-MS Data Correlation Analysis
Source: Foods. 2022 Sep 16;11(18):2880. doi: 10.3390/foods11182880 (PMC9498329; doi:10.3390/foods11182880)
Supplement: Supplementary file 1 [file foods-11-02880-s001.zip › foods-1898447-supplementary.pdf]

Supplemental information:

Table S1. Expert sensory and consumer acceptance evaluation of six oolong tea samples.

Table S2. The variables of the final step in the multiple regression linear model of consumer acceptance and its parameter estimation.

Table S3. The optimal multiple linear regression model equation.

Table S4. Parameter estimates, probability values and odds ratios of the influence of consumers' purchase intention.

Table S5. Stable response values of electronic nose sensors for six oolong tea samples.

Table S6. Adonis analysis results of adaptive grouping in PCA analysis of electronic nose data

Table S7. Adonis analysis results of sample grouping in PCoA analysis of HS-SPME-GC-MS data

Table S8. Adonis analysis results of suitability grouping in PCoA analysis of HS-SPME-GC-MS data of different samples

Table S9 Relative content of aroma compounds in HS-SPME-GC-MS analysis of six oolong tea samples.

Table S10 Spearman correlation coefficient of between electronic nose data, HS-SPME-GC-MS data, and sensory data in six tea cultivars.

Table S1 Expert sensory and consumer acceptance evaluation of six oolong tea samples

Cultivars	Score of experts	Score of consumers	Aroma	Total Score	Suitability
FD	78.5±0.46d	5.37±0.87d	green	77.74±0.22d	Green tea, Black tea, White Tea
YS	79.17±0.35d	5.45±1.53cd	green	79.02±0.42d	Green tea, Black tea
WNZ	81.83±1.24c	5.25±0.75d	green	81.33±0.67c	Green tea
MZ	85.83±0.99b	6.52±1b	Flavour	85.16±0.68b	Oolong tea, Green tea, Black tea
HD	89.17±1.24a	7.57±1.01a	Heavy flavour	86.48±0.98ab	Oolong tea, Green tea, Black tea
TGY	89.17±0.35a	5.86±1.66c	Heavy flavour	86.66±0.33a	Oolong tea, Green tea

Note: The significance of difference was calculated *via* one-way analysis of variance (ANOVA) at $P < 0.05$; the relative content of each compound was expressed as average ± standard error of the mean (n=3)

Table S2 The variables of the final step in the multiple regression linear model of consumer acceptance and its parameter estimation

	WNZ		YS		FD		HD		MZ		TGY	
	Parameter estimates	Pr > χ^2	Parameter estimates	Pr > χ^2	Parameter estimates	Pr > χ^2	Parameter estimates	Pr > χ^2	Parameter estimates	Pr > χ^2	Parameter estimates	Pr > χ^2
X_1	1.56509	<.0001			0.32308	0.0002	0.47028	<.0001				
X_2												
X_3	-0.6822	0.035	0.67483	<.0001	0.70018	<.0001	0.32085	0.0004	0.90357	<.0001	0.42598	<.0001
X_4											0.31507	<.0001
X_{11}												
X_{22}												
X_{33}									-0.04242	0.0066		
X_{44}												
X_{12}	-0.17756	0.0051	0.04151	<.0001					0.05555	<.0001	0.03396	0.0008
X_{13}												
X_{14}												
X_{23}	0.19058	0.001										

X_{24}	0.02997	0.0006
X_{34}		

Table S3 The optimal multiple linear regression model equation

Cultivars	Model equation	F Value	R^2	Adj R^2
WNZ	Y=1.56509X1-0.6822X3-0.17756X12+0.19058x23	973.27	0.9853	0.9843
YS	Y=0.67483X3+0.04151X12	1800.94	0.9816	0.981
FD	Y=0.32308X1+0.70018X3	1600.13	0.9836	0.9831
HD	Y=0.47028X1+0.32085X3+0.02997X24	1716.22	0.9887	0.9881
MZ	Y=0.90357X3-0.04242X33+0.05555X12	1108.67	0.9826	0.9817
TGY	Y=0.42598X3+0.31507X4+0.03396X12	1197.18	0.9838	0.983

Note: For parameter estimates, the estimated parameter is the coefficient of the independent variable in the model equation, describing the influence of the independent variable on the dependent variable; Pr > $\chi 2$ is the probability value of the estimated parameter ($p<0.05$ level). The REG algorithm in SAS 3.0 software was used for multiple regression analysis of the relationship between each endoplasmic factor and the overall acceptance index (overall evaluation). The overall evaluation index was used as the dependent variable (Y), and the four indicators (aroma, soup color, taste,

and infused leaf) as the original independent variables (X_1, X_2, X_3, X_4). Due to the interaction between the indicators and the original independent variables, we set X_{11}, X_{22}, X_{33}, X_{44}, X_{12}, X_{13}, X_{14}, X_{23}, X_{24}, and X_{34} to represent the interaction between the indicators as binary independent variables ($X_{11}=X_1*X_1$, $X_{12}=X_1*X_2$, $X_{13}=X_1*X_3$, $X_{14}=X_1*X_4$, $X_{22}=X_2*X_2$, $X_{23}=X_2*X_3$, $X_{24}=X_2*X_4$, $X_{33}=X_3*X_3$, $X_{34}=X_3*X_4$, $X_{44}=X_4*X_4$), and the original independent variables and binary independent variables were used for multiple regression analysis through the stepwise selection method to filter out the optimal indicators.

Table S4 Parameter estimates, probability values and odds ratios of the influence of consumers' purchase intention

		Aroma	Liquor color	Taste	Infused leaf
WNZ	Parameter estimates	-1.2141	0.3147	1.9279	0.8372
	Pr > χ2	0.1583	0.6299	0.0724	0.2999
	odds ratio	0.297	1.37	6.875	2.31
YS	Parameter estimates	0.6146	-0.2586	0.7226	0.3123
	Pr > χ2	0.1051	0.5581	0.1475	0.5051
	odds ratio	1.849	0.772	2.06	1.367
FD	Parameter estimates	-0.9758	0.2847	2.0799	-0.2165
	Pr > χ2	0.1662	0.7632	0.0256	0.7313
	odds ratio	0.377	1.329	8.004	0.805
HD	Parameter estimates	0.6796	0.3962	0.2043	0.3138
	Pr > χ2	0.0276	0.2031	0.4703	0.1661
	odds ratio	1.973	1.486	1.227	1.369
MZ	Parameter estimates	0.2468	0.4449	0.7147	0.1746
	Pr > χ2	0.5309	0.2199	0.0978	0.5892
	odds ratio	1.28	1.56	2.044	1.191
TGY	Parameter estimates	0.8962	-0.0164	0.4997	0.35
	Pr > χ2	0.0456	0.9778	0.2832	0.5414
	odds ratio	2.45	0.984	1.648	1.419

Note: For parameter estimates, the estimated parameter is the coefficient of the independent variable in the model equation, describing the influence of the independent variable on the dependent variable; Pr > χ2 is the probability value of the estimated parameter ($p<0.05$ level); since consumer purchase intention is a binary variable, we used logistic regression analysis to analyze the influence of four variables (aroma, soup color, taste, and infused leaf) on consumer purchase intention. A consumer's purchase intention score greater than "3" is defined as willing to buy, and a

score less than or equal to "3" is defined as unwilling to buy. Cells marked in red represent significant results.

Table S5 Stable response values of sensors in electronic nose analysis of six oolong tea samples

	LY2/LG	LY2/G	LY2/AA	LY2/GH	LY2/gCTL	LY2/gCT	T30/1	P10/1	P10/2	P40/1	T70/2	PA/2	P30/1	P40/2	P30/2	T40/2	T40/1	TA/2
**FD_1**	**0.03875**	**-0.091**	**-0.037**	**-0.0807**	**-0.0963**	**-0.1898**	**0.78651**	**0.83547**	**0.62152**	**0.7281**	**0.74935**	**0.89496**	**0.87681**	**0.59597**	**0.74041**	**0.58605**	**0.54766**	**0.67552**
**FD_2**	**0.04224**	**-0.0922**	**-0.0373**	**-0.0825**	**-0.0905**	**-0.1961**	**0.80651**	**0.85289**	**0.6394**	**0.74818**	**0.76868**	**0.9027**	**0.88856**	**0.61819**	**0.74104**	**0.60535**	**0.58769**	**0.70412**
**FD_3**	**0.04049**	**-0.0868**	**-0.0359**	**-0.0778**	**-0.0925**	**-0.1883**	**0.79755**	**0.84929**	**0.6345**	**0.74358**	**0.75789**	**0.89773**	**0.88421**	**0.60773**	**0.72395**	**0.60161**	**0.57756**	**0.70667**
**FD_4**	**0.04136**	**-0.0931**	**-0.0378**	**-0.0826**	**-0.1046**	**-0.1992**	**0.79793**	**0.84695**	**0.62181**	**0.73892**	**0.75808**	**0.89351**	**0.88439**	**0.60354**	**0.7343**	**0.59955**	**0.57108**	**0.67917**
**HD_1**	**0.0488**	**-0.0465**	**-0.0285**	**-0.0427**	**-0.0525**	**-0.0764**	**0.66568**	**0.71462**	**0.52922**	**0.60614**	**0.60691**	**0.74615**	**0.779**	**0.51008**	**0.65056**	**0.475**	**0.4924**	**0.61364**
**HD_2**	**0.04465**	**-0.0473**	**-0.0286**	**-0.0453**	**-0.0568**	**-0.0799**	**0.63651**	**0.73112**	**0.56133**	**0.63572**	**0.58437**	**0.77524**	**0.76173**	**0.49592**	**0.66185**	**0.46859**	**0.58288**	**0.66604**
**HD_3**	**0.04672**	**-0.0497**	**-0.0324**	**-0.0449**	**-0.0767**	**-0.0849**	**0.63017**	**0.72682**	**0.55052**	**0.62965**	**0.57609**	**0.75284**	**0.75013**	**0.48704**	**0.63071**	**0.45702**	**0.52491**	**0.64176**
**HD_4**	**0.04569**	**-0.0446**	**-0.0276**	**-0.0411**	**-0.0646**	**-0.0901**	**0.66283**	**0.74873**	**0.56639**	**0.64895**	**0.61101**	**0.7931**	**0.78023**	**0.50601**	**0.65218**	**0.47802**	**0.55732**	**0.659**
**MZ_1**	**0.02628**	**-0.0342**	**-0.0242**	**-0.0309**	**-0.0457**	**-0.0653**	**0.58353**	**0.69644**	**0.52297**	**0.60362**	**0.5243**	**0.72126**	**0.70897**	**0.44747**	**0.56539**	**0.41983**	**0.50989**	**0.61464**
**MZ_2**	**0.02906**	**-0.0362**	**-0.0252**	**-0.0324**	**-0.0587**	**-0.0596**	**0.56248**	**0.6797**	**0.50786**	**0.5894**	**0.50315**	**0.70084**	**0.68907**	**0.42758**	**0.52733**	**0.40026**	**0.46956**	**0.60338**
**MZ_3**	**0.02767**	**-0.0347**	**-0.0226**	**-0.0323**	**-0.0508**	**-0.0729**	**0.65728**	**0.74055**	**0.54834**	**0.63922**	**0.59824**	**0.75645**	**0.76413**	**0.49622**	**0.6146**	**0.46688**	**0.56456**	**0.67297**
**MZ_4**	**0.02837**	**-0.0353**	**-0.023**	**-0.0322**	**-0.0584**	**-0.0733**	**0.66931**	**0.74421**	**0.54686**	**0.64096**	**0.60851**	**0.75502**	**0.77183**	**0.50448**	**0.61394**	**0.47315**	**0.50725**	**0.64571**
**TGY_1**	**0.02852**	**-0.0401**	**-0.0183**	**-0.0349**	**-0.0491**	**-0.0857**	**0.65613**	**0.75439**	**0.52585**	**0.63434**	**0.58972**	**0.77708**	**0.77523**	**0.48183**	**0.54234**	**0.47225**	**0.51667**	**0.60729**
**TGY_2**	**0.03365**	**-0.0483**	**-0.0222**	**-0.041**	**-0.0465**	**-0.1028**	**0.71474**	**0.78829**	**0.55668**	**0.66803**	**0.65639**	**0.82264**	**0.81907**	**0.52959**	**0.62385**	**0.5153**	**0.52628**	**0.63793**
**TGY_3**	**0.03108**	**-0.0516**	**-0.0206**	**-0.0443**	**-0.0468**	**-0.1061**	**0.69789**	**0.78347**	**0.55861**	**0.66696**	**0.63846**	**0.82553**	**0.81229**	**0.52072**	**0.59862**	**0.50923**	**0.55422**	**0.654**

**TGY_4**	**0.03236**	**-0.0496**	**-0.0206**	**-0.0436**	**-0.0491**	**-0.1049**	**0.70686**	**0.78834**	**0.56176**	**0.67426**	**0.64684**	**0.82353**	**0.81535**	**0.52699**	**0.60615**	**0.51259**	**0.53314**	**0.66064**
**WNZ_1**	**0.01326**	**-0.0655**	**-0.0293**	**-0.0576**	**-0.0678**	**-0.1307**	**0.7056**	**0.77331**	**0.56953**	**0.66239**	**0.6617**	**0.84461**	**0.82178**	**0.5237**	**0.69692**	**0.5098**	**0.52242**	**0.62118**
**WNZ_2**	**0.01617**	**-0.0683**	**-0.0297**	**-0.0613**	**-0.08**	**-0.1361**	**0.72274**	**0.78917**	**0.57688**	**0.67996**	**0.67618**	**0.84945**	**0.83104**	**0.53741**	**0.68481**	**0.52862**	**0.51165**	**0.64257**
**WNZ_3**	**0.01471**	**-0.0607**	**-0.0265**	**-0.0531**	**-0.0619**	**-0.1186**	**0.69388**	**0.77332**	**0.574**	**0.66451**	**0.64263**	**0.82457**	**0.81098**	**0.51564**	**0.64416**	**0.50149**	**0.51277**	**0.64023**
**WNZ_4**	**0.01544**	**-0.0664**	**-0.0316**	**-0.0583**	**-0.0896**	**-0.1457**	**0.76039**	**0.80978**	**0.59404**	**0.70229**	**0.71624**	**0.86629**	**0.85654**	**0.56802**	**0.72609**	**0.55004**	**0.55849**	**0.66157**
**YS_1**	**0.03037**	**-0.0573**	**-0.0244**	**-0.0502**	**-0.0681**	**-0.1322**	**0.75501**	**0.8152**	**0.58369**	**0.70068**	**0.70164**	**0.85607**	**0.84544**	**0.56497**	**0.6691**	**0.55457**	**0.55171**	**0.66068**
**YS_2**	**0.02999**	**-0.0529**	**-0.0239**	**-0.0464**	**-0.0713**	**-0.1235**	**0.74811**	**0.81452**	**0.58438**	**0.70061**	**0.69195**	**0.85478**	**0.83896**	**0.56226**	**0.64978**	**0.55115**	**0.55559**	**0.67614**
**YS_3**	**0.03018**	**-0.0603**	**-0.0283**	**-0.0527**	**-0.0811**	**-0.1361**	**0.77217**	**0.825**	**0.58698**	**0.70678**	**0.72195**	**0.86017**	**0.85744**	**0.57903**	**0.6842**	**0.56916**	**0.54066**	**0.6581**
**YS_4**	**0.03018**	**-0.0568**	**-0.0255**	**-0.0498**	**-0.0735**	**-0.1306**	**0.75843**	**0.81824**	**0.58502**	**0.70269**	**0.70518**	**0.85701**	**0.84728**	**0.56875**	**0.66769**	**0.55829**	**0.54932**	**0.66497**

Table S6 Adonis analysis results of adaptive grouping in PCA analysis of electronic nose data

	Df	SumOfSqs	R^2	F	Pr(>F)
Classification	1	0.77346	0.57068	29.244	0.001***
Residual	22	0.58188	0.42932		
Total	23	1.35534	1.00000		

Table S7 Adonis analysis results of sample grouping in PCoA analysis of HS-SPME-GC-MS data

	Df	SumOfSqs	R^2	F	Pr(>F)
Classification	1	0.070942	0.51893	17.259	0.001***
Residual	16	0.065766	0.48107		
Total	17	0.136707	1.00000		

Table S8 Adonis analysis results of suitability grouping in PCoA analysis of HS-SPME-GC-MS data of different samples

	Df	SumOfSqs	R^2	F	Pr(>F)
Sample	5	0.99962	0.97109	80.612	0.001***
Residual	12	0.02976	0.02891		
Total	17	1.02938	1.00000		

Table S9 Relative content of aroma compounds in HS-SPME-GC-MS analysis of six oolong tea samples.

Name	type	HD	MZ	TGY	FD	WNZ	YS	p	p_value	RT	Experimental RI	Reference RI	CAS	Descriptors in website	Descriptors in reference	references
Benzaldehyde	AADVs	4.995±0.572	4.318±0.516	4.836±0.097	3.634±0.588	3.621±0.855	2.519±0.208	**	0.001268	7.58	938	962	100-52-7	strong sharp sweet bitter almond cherry	almond	[40]
1-Heptanol	FADVs	5.991±0.118	3.154±0.036	6.753±1.021	0.51±0.422	6.46±0.25	5.276±0.124	NA	0.353837	7.78	947	970	111-70-6	musty leafy violet herbal green sweet woody peony	plant	[40]
1-Octen-3-ol	FADVs	5.206±0.936	1.875±0.335	2.031±0.05	1.282±0.296	3.494±0.496	1.226±0.043	NA	0.057631	7.89	952	982	3391-86-4	mushroom earthy green oily fungal raw chicken	mushroom	[40]
6-methyl-5-Hepten-2-one	CDVs	49.646±6.357	22.978±3.741	29.408±0.725	12.767±1.278	15.88±2.578	10.786±0.518	**	0.000349	8.18	965	986	110-93-0	citrus	green bean	[41]
2-Pentylfuran	others	21.522±4.13	18.608±2.348	22.664±1.836	12.522±2.346	9.481±0.2	6.687±0.501	**	0.000349	8.38	975	979	3777-69-3	fruity	floral	[41]
Octanal	others	2.359±0.224	3.09±0.091	2.475±0.162	0.405±0.066	0.606±0.021	1.3±0.08	**	0.000349	8.74	991	1003	124-13-0	Aldehydic, waxy, citrus orange with a green peely nuance	citrus, aldehydic, peel , green	[42]
(E,E)-2,4-Heptadienal	FADVs	7.438±0.906	7.906±0.995	1.37±0.007	3.677±0.712	2.343±0.434	1.376±0.104	NA	0.085089	8.97	1001	1012	4313-03-5	Fatty, green, oily, aldehydic with a vegetative nuance	fatty	[41]
Phenylacetaldehyde	AADVs	4.26±0.32	3.094±0.103	5.756±0.023	1.729±0.135	3.461±0.585	3.019±0.401	*	0.015169	10.13	1030	1045	122-78-1	Honey, floral rose, sweet, powdery, fermented, chocolate with a slight earthy nuance	floral, rose	[40]
β-Ocimene	VTs	122.544±22.69	129.683±17.943	61.565±3.187	40.601±9.523	11.841±2.938	10.597±1.212	**	0.000349	10.29	1034	1037	13877-91-3	floral,Tropical, green, terpy and woody with vegetable nuances	slightly floral	[43]
(Z)-Furan linalool oxide	VTs	77.707±0.929	73.05±7.885	33.41±4.645	28.022±1.544	38.978±5.292	12.479±0.5	**	0.005411	11.26	1059	1074	5989-33-3	earthy floral sweet woody	earthy floral , sweet woody	[40]
(E)-Furan linalool oxide	VTs	51.225±9.371	20.778±2.187	14.137±2.66	14.519±0.801	8.476±0.44	5.448±0.297	**	0.00172	11.94	1076	1086	34995-77-2	floral	floral	[40]
linalool	VTs	267.016±35.698	314.73±57.3	103.264±21.521	74.465±3.253	112.028±11.256	17.799±0.312	**	0.00172	12.51	1090	1099	78-70-6	floral，Citrus, orange, floral, terpy, waxy and rose	citrus,floral	[41]
1,5,7-Octatrien-3-ol, 3,7-dimethyl-	VTs	207.888±40.014	92.287±11.628	31.763±9.528	127.17±21.027	56.931±0.648	3.671±0.029	NA	0.23323	12.66	1094	1107	29957-43-5	mouldy	ripe fruit	[44]
Phenylethyl alcohol	AADVs	23.69±2.611	26.184±3.019	11.596±2.236	11.313±1.732	6.86±0.318	12.308±1.846	**	0.005411	12.98	1102	1116	1960, 12, 8	floral , sweet floral fresh bready rose honey	sweet floral，fresh rose honey	[40]
(3E)-4,8-Dimethyl-1,3,7-nonatriene	VTs	51.028±8.093	68.657±8.507	59.225±4.24	30.621±6.026	9.674±0.219	18.078±1.602	**	0.000349	13.15	1105	1116	19945-61-0	NA	floral scent	[45]

Cosmene	VTs	12.785±1.84	5.516±0.981	5.432±0.331	5.644±1.185	2.942±0.077	1.023±0.15	**	0.007077	13.89	1120	1131	460-01-5	NA	NA	
Benzyl nitrile	AADVs	14.943±1.027	10.821±0.895	4.085±0.16	9.577±2.553	5.43±0.566	6.077±0.718	NA	0.353837	14.15	1125	1144	140-29-4	NA	NA	
(R,S)-5-Ethyl-6-methyl-3E-hepten-2-one	AADVs	35.633±3.095	11.863±0.596	44.964±8.105	7.235±0.113	6.852±1.211	6.378±0.312	**	0.000349	14.39	1130	1144	57283-79-1	NA	special aroma	[46]
(E)-Pyranoid linalool oxide	VTs	39.437±7.584	18.455±1.516	17.363±0.093	13.709±2.289	10.627±1.133	5.96±0.264	**	0.000349	15.87	1159	1173	39028-58-5	woody	woody	[40]
Linalool oxide pyranoside	VTs	38.651±4.976	10.329±2.003	26.552±2.595	17.145±0.9	5.354±0.064	7.88±0.565	**	0.005411	16.11	1164	1178	14049-11-7	floral honey	earthy	[21]
(E)-2,6-Dimethyl-3,7-octadiene-2,6-diol	VTs	21.362±1.752	4.413±0.234	2.807±0.45	3.9±0.031	17.076±4.211	0.469±0.08	NA	0.26969	16.85	1178	1190	13741-21-4	NA	sweet,citrus	[47]
Methyl salicylate	AADVs	53.49±6.521	31.148±6.064	7.235±1.817	5.486±0.06	7.924±2.86	4.002±0.382	**	0.004107	16.95	1180	1192	119-36-8	minty , Sweet, salicylate, root beer, wintergreen, aromatic, slightly phenolic and camphoreous	Sweet , wintergreen , romatic, phenolic	[40]
Butanoic acid, hexyl ester	FADVs	7.528±0.545	7.377±1.463	12.504±0.015	5.064±0.074	3.695±0.287	2.766±0.216	**	0.000349	17.1	1183	1192	2639-63-6	green , green sweet fruity apple waxy soapy	fruity,andvegetative	[48]
α-Terpineol	VTs	3.833±0.171	5.445±0.721	1.351±0.078	3.378±0.054	2.114±0.305	0.62±0.026	*	0.046945	17.2	1185	1189	98-55-5	terpenic, pine terpenic lilac citrus woody floral	lemon citrus , floral	[40]
Dodecane	others	5.743±0.929	3.874±0.407	30.745±2.315	4.512±0.328	6.124±0.924	2.632±0.226	NA	0.14512	17.63	1194	1200	112-40-3	NA	NA	
Decanal	others	11.387±1.72	7.811±1.384	8.074±0.904	3.872±0.606	4.873±0.661	2.518±0.043	**	0.000349	17.86	1198	1206	112-31-2	aldehydic , weet aldehydic waxy orange peel citrus floral	vegetal, fruity, flowery	[49]
(E,E)-2,4-Nonadienal	others	4.149±0.489	3.179±0.542	3.583±0.156	1.779±0.176	2.195±0.192	1.184±0.132	**	0.000349	18.28	1206	1216	5910-87-2	fatty, fatty melon waxy green violet leaf cucumber fruit tropical fruit chicken fat	baked bread, peanut	[49]
Linalyl formate	VTs	4.989±0.315	5.935±0.917	2.034±0.254	2.124±0.025	1.878±0.208	0.829±0.043	**	0.005411	18.8	1215	1215	115-99-1	herbal , citrus herbal bergamot lavender soapy fatty green woody	sweet odor	[50]
(Z)-3-Hexenyl-α-methylbutyrate	FADVs	47.6±4.337	49.716±7.811	6.886±0.062	27.537±5.694	16.204±1.764	9.592±0.83	NA	0.23323	19.21	1222	1234	53398-85-9	green , fresh green apple sweet fruity pear	herbal, sweet	[51]
(Z)-3-Hexenyl isovalerate	FADVs	15.334±2.074	24.667±4.362	9.263±0.256	18.965±2.673	14.168±3.237	4.69±0.588	NA	0.401542	19.51	1227	1238	35154-45-1	green , fresh apple green apple fruity tropical pineapple	fruity green, waxy pear, winey	[40]
Geraniol	VTs	60.763±7.452	110.334±19.314	19.351±2.07	33.67±1.182	20.109±2.432	5.943±0.546	*	0.024341	20.28	1241	1255	106-24-1	floral , sweet floral fruity rose waxy citrus	sweet floral, fruity rose waxy, citrus	[40]
(Z)-2-Decenal	others	19.263±1.802	10.659±1.37	9.747±0.324	5.469±1.107	5.448±0.63	3.045±0.249	**	0.000349	20.95	1253	1252	2497-25-8	fatty, tallow fatty	fruity or fat odor	[52]

Indole	AADVs	191.251±15.395	100.832±12.531	82.382±0.908	47.926±1.621	32.312±4.571	41.379±1.18	**	0.000349	22.37	1278	1295	120-72-9	animal, pungent floral naphthyl fecal animal musty	sour fruit	[53]
Methyl geranate	others	4.525±0.257	4.254±0.008	1.639±0.007	2.13±0.152	1.03±0.077	1.401±0.03	**	0.005411	24.41	1313	1323	2349-14-6	NA	sweet candy	[54]
α-Cubebene	VTs	18.816±3.553	4.275±0.144	4.24±0.713	4.471±0.43	1.976±0.329	0.912±0.138	**	0.00919	25.88	1338	1351	17699-14-8	herbal, herbal waxy	wax, herb,ripe fruit	[55]
(E)-2-Undecenal	others	16.934±1.098	8.115±1.421	11.859±2.321	4.489±0.761	4.616±0.892	3.465±0.382	**	0.000349	27.11	1358	1365	53448-07-0	fruity, fresh fruity citrus orange peel	Fresh fragrance, cabbage, cabbage, copper	[56]
(Z)-3-Hexenyl hexanoate	FADVs	317.295±39.426	168.079±26.335	94.47±17.301	116.897±14.308	92.21±1.337	52.673±6.39	**	0.00919	28.21	1377	1386	31501-11-8	green, fruity green waxy pear winey tropical grassy pineapple	fruity green, waxy pear, winey	[40]
(Z)-3-Hexenyl (Z)-3-hexenoate	FADVs	63.337±7.789	30.419±1.471	41.91±7.93	5.975±0.597	7.018±0.692	6.132±0.632	**	0.000349	28.41	1380	1389	61444-38-0	green, green tomato leaf pear melon metallic fennel tropical	Passion fruit	[57]
Hexanoic acid, hexyl ester	FADVs	67.922±6.959	33.906±2.603	41.081±6.914	59.006±4.562	48.296±7.545	10.389±0.443	NA	0.691102	28.55	1382	1384	6378-65-0	green, herbal fresh green grassy vegetable fruity	Grass	[58]
(Z)-Jasmone	FADVs	34.521±4.008	22.828±3.482	48.314±0.885	16.363±2.117	10.992±0.896	13.977±1.129	**	0.000349	28.62	1384	1394	488-10-8	floral, woody herbal floral spicy jasmin celery	fruity rose, honey tobacco, sweet	[40]
(E)-2-Hexenyl hexanoate	FADVs	23.673±2.315	13.471±2.327	36.524±2.946	10.842±1.148	36.023±7.015	7.791±0.729	NA	0.102345	28.71	1385	1391	53398-86-0	green, green natural cognac herbal waxy clean	green	[59]
β-Caryophyllen	VTs	30.431±4.077	14.33±1.695	15.892±0.095	18.55±1.865	4.038±0.092	2.031±0.135	*	0.046945	29.97	1409	1419	87-44-5	spicy, sweet woody spicy clove dry	woody and spicy	[60]
α-Ionone	FADVs	10.65±1.389	5.636±0.429	12.31±1.575	5.887±0.625	6.062±0.639	5.468±0.312	*	0.03051	30.16	1414	1426	127-41-3	floral, sweet woody floral violet orris tropical fruity	sweet woody, floral violet, orris tropical, fruity	[40]
γ-Elemene	VTs	7.263±1.298	8.165±1.221	8.807±1.557	3.66±0.394	2.241±0.21	1.833±0.048	**	0.000349	30.54	1423	1433	29873-99-2	NA	NA	
β-Phenylethyl butyrate	AADVs	6.908±1.303	8.994±1.768	1.992±0.199	5.431±0.203	1.354±0.238	3.633±0.365	NA	0.057631	30.98	1433	1447	103-52-6	floral, musty sweet floral yeasty strawberry	fruity, sweet	[40]
Isoeugenol	others	9.893±0.517	9.464±0.387	0±0	3.99±0.073	8.498±0.596	2.123±0.136	NA	0.232264	31.2	1440	1450	97-54-1	spicy, sweet spicy clove woody allspice carnation floral	floral, spicy	[61]
(E)-Geranylacetone	CDVs	50.619±7.127	22.795±2.836	29.766±3.948	17.91±2.485	22.746±1.911	19.925±0.673	**	0.002316	31.43	1444	1435	3796-70-1	floral,fresh green fruity waxy rose woody magnolia tropical	Honey, floral, spicy, sweet	[62]
Humulene	VTs	10.918±1.808	4.502±0.339	9.69±1.609	3.063±0.141	4.328±0.143	1.47±0.09	**	0.000929	31.56	1447	1454	6753-98-6	woody	woody, musty (in beer)	[63]
(E)-β-Famesene	VTs	34.831±1.657	18.412±3.183	20.624±0.593	10.868±0.951	8.395±0.2	5.228±0.081	**	0.000349	31.7	1451	1457	18794-84-8	woody,woody citrus herbal sweet	Wood, citrus, sweet	[64]

(E)-2-Dodecenal	others	5.972±0.385	2.595±0.374	10.496±0.344	2.265±0.062	2.784±0.072	2.194±0.213	**	0.007077	32.23	1464	1468	20407-84-5	herbal, citrus metallic mandarin orange waxy aldehydic	paperboard	[65]
β-Ionone	CDVs	50.133±7.248	28.747±3.185	51.626±4.696	21.315±2.959	22.586±1.047	20.638±0.363	**	0.000349	32.58	1472	1491	79-77-6	floral,dry powdery floral woody orris berry seedy	sweet fruity , woody berry , floral seedy	[40]
(Z,E)-α-Farnesene	VTs	80.357±6.414	36.165±3.406	64.668±7.148	26.101±0.365	27.531±0.814	15.328±0.539	**	0.000349	33.2	1487	1491	26560-14-5	NA	NA	
α-Muurolene	VTs	32.145±4.023	7.807±1.157	16.3±0.433	9.468±1.582	3.027±0.135	1.589±0.248	**	0.003095	33.35	1491	1499	10208-80-7	NA	NA	
α-Farnesene	VTs	1310.571±67.069	772.189±116.822	613.723±21.838	626.814±40.145	631.589±79.248	328.155±36.369	**	0.00919	33.73	1501	1508	502-61-4	woody,citrus herbal lavender bergamot myrrh neroli green	fruity,wood, sweet, floral, herb	[66]
γ-Cadinene	VTs	19±2.324	0±0	12.084±0.788	6.798±0.585	0±0	1.613±0.022	NA	0.105459	33.85	1504	1513	39029-41-9	woody,herbal woody	NA	[21]
δ-Cadinene	VTs	132.843±8.435	28.198±3.893	44.899±7.918	40.784±6.194	11.34±2.128	3.965±0.622	*	0.011849	34.07	1511	1524	483-76-1	herbal,thyme herbal woody dry	herbal, woody	[67]
(E)-Nerolidol	VTs	499.908±100.29	536.389±106.633	185.131±10.677	271.212±10.693	205.391±23.341	155.583±3.465	*	0.037977	35.54	1558	1564	7212-44-4	floral, floral green waxy citrus woody	floral green，citrus woody， waxy	[40]
(Z)-3-Hexenyl benzoate	AADVs	69.185±6.964	68.167±12.479	52.654±5.564	56.922±0.175	33.778±1.854	38.96±1.8	**	0.005411	35.77	1565	1570	25152-85-6	green, green sweet oily floral spicy fruity	pear-like	[58]
Nerolidol	VTs	41.799±5.71	20.466±3.095	45.321±3.563	24.826±1.888	41.061±7.426	10.851±0.253	NA	0.171098	35.93	1571	1564	40716-66-3	floral, floral green citrus woody waxy	Rose-like	[54]
Benzoic acid, hexyl ester	AADVs	17.582±0.826	12.13±2.237	11.815±0.682	17.429±0.035	11.995±2.107	7.205±0.713	NA	0.353837	36.03	1574	1580	6789-88-4	balsamic, fresh balsamic sappy clean woody	sweet fruit	[68]
E-2-Hexenyl benzoate	AADVs	7.095±1.085	3.165±0.525	6.524±0.908	4.551±0.016	7.737±1.319	4.191±0.517	NA	0.894626	36.26	1581	1588	76841-70-8	NA	NA	
Geranyl isovalerate	AADVs	9.1±1.111	3.279±0.336	7.237±0.193	2.85±0.31	3.089±0.488	1.739±0.249	**	0.001268	36.72	1596	1606	109-20-6	fruity, green fruity apple blueberry pineapple	NA	
T-Muurolol	VTs	26.752±1.516	4.305±0.354	7.77±0.745	4.297±0.086	1.235±0.156	0.885±0.294	**	0.001268	37.85	1636	1642	19912-62-0	herbal, herbal spicy honey	NA	
α-Cadinol	VTs	45.47±4.808	9.332±1.553	16.539±1.209	10.247±1.053	7.162±0.357	4.161±0.609	**	0.002316	38.17	1650	1653	481-34-5	herbal, herbal woody	spicy	[69]

Note: p: significant results based on Kruskal-Wallis test; "*" indicates $p<0.05$; "**" indicates $p<0.01$, and "NA" indicates $p>0.05$. Experimental RI: retention index in the research. Reference RI: retention index in the NIST 2014 database. CAS: the CAS number in the NIST 2014 database. Descriptors in website: the Descriptors in the website: http://www.thegoodscentscompany.com.

Table S10 Spearman correlation coefficient of between electronic nose data, HS-SPME-GC-MS data, and sensory data in six tea cultivars.

	P	TYPE	LY2G	LY2AA	LY2GH	LY2gCTL	LY2gCT	T301	P101	P102	P401	T702	PA2	P301	P402	P302	T402	TA2	Expert	Consumer
Benzaldehyde	**	AADVs	0.6	0.314286	0.542857	0.6	0.6	-0.65714	-0.65714	-0.6	-0.65714	-0.65714	-0.65714	-0.65714	-0.65714	-0.54286	-0.65714	-0.37143	0.811679	0.771429
6-methyl-5-Hepten-2-one	**	CDVs	0.657143	0.371429	0.6	0.657143	0.657143	-0.77143	-0.77143	-0.71429	-0.77143	-0.77143	-0.77143	-0.77143	-0.77143	-0.6	-0.77143	-0.54286	0.927634	0.714286
2-Pentylfuran	**	others	0.542857	0.485714	0.6	0.714286	0.542857	-0.6	-0.6	-0.65714	-0.6	-0.6	-0.6	-0.6	-0.6	-0.6	-0.6	-0.48571	0.811679	0.657143
Octanal	**	others	0.942857	0.885714	1	0.942857	0.942857	-0.88571	-0.88571	-0.94286	-0.88571	-0.88571	-0.88571	-0.88571	-0.88571	-1	-0.88571	-0.82857	0.753702	0.771429
Phenylacetaldehyde	*	AADVs	0.485714	0.542857	0.542857	0.714286	0.485714	-0.6	-0.6	-0.65714	-0.6	-0.6	-0.6	-0.6	-0.6	-0.54286	-0.6	-0.6	0.927634	0.428571
β-Ocimene	**	VTs	0.771429	0.371429	0.714286	0.6	0.771429	-0.82857	-0.82857	-0.77143	-0.82857	-0.82857	-0.82857	-0.82857	-0.82857	-0.71429	-0.82857	-0.6	0.637748	0.771429
(Z)-Furan linalool oxide	**	VTs	0.657143	0.085714	0.485714	0.371429	0.657143	-0.82857	-0.82857	-0.65714	-0.82857	-0.82857	-0.82857	-0.82857	-0.82857	-0.48571	-0.82857	-0.54286	0.695725	0.6
(E)-Furan linalool oxide	**	VTs	0.542857	-0.02857	0.371429	0.257143	0.542857	-0.6	-0.6	-0.42857	-0.6	-0.6	-0.6	-0.6	-0.6	-0.37143	-0.6	-0.2	0.463817	0.714286
linalool	**	VTs	0.714286	0.2	0.6	0.428571	0.714286	-0.88571	-0.88571	-0.77143	-0.88571	-0.88571	-0.88571	-0.88571	-0.88571	-0.6	-0.88571	-0.71429	0.60876	0.542857
Phenylethyl alcohol	**	AADVs	0.885714	0.542857	0.771429	0.6	0.885714	-0.71429	-0.71429	-0.6	-0.71429	-0.71429	-0.71429	-0.71429	-0.71429	-0.77143	-0.71429	-0.37143	0.434828	0.885714
(3E)-4,8-Dimethyl-1,3,7-nonatriene	**	VTs	0.771429	0.657143	0.828571	0.771429	0.771429	-0.71429	-0.71429	-0.77143	-0.71429	-0.71429	-0.71429	-0.71429	-0.71429	-0.82857	-0.71429	-0.6	0.579771	0.771429
Cosmene	**	VTs	0.257143	-0.25714	0.085714	0.028571	0.257143	-0.31429	-0.31429	-0.14286	-0.31429	-0.31429	-0.31429	-0.31429	-0.31429	-0.08571	-0.31429	0.085714	0.289886	0.542857
(R,S)-5-Ethyl-6-methyl-3E-hepten-2-one	**	CDVs	0.542857	0.485714	0.6	0.714286	0.542857	-0.6	-0.6	-0.65714	-0.6	-0.6	-0.6	-0.6	-0.6	-0.6	-0.6	-0.48571	0.811679	0.657143
(E)-Pyranoid linalool oxide	**	VTs	0.714286	0.257143	0.6	0.542857	0.714286	-0.77143	-0.77143	-0.65714	-0.77143	-0.77143	-0.77143	-0.77143	-0.77143	-0.6	-0.77143	-0.42857	0.724714	0.828571
Linalool oxide pyranoside	**	VTs	0.371429	0.2	0.314286	0.428571	0.371429	-0.31429	-0.31429	-0.25714	-0.31429	-0.31429	-0.31429	-0.31429	-0.31429	-0.31429	-0.31429	0.028571	0.579771	0.714286
Methyl salicylate	**	AADVs	0.657143	0.085714	0.485714	0.371429	0.657143	-0.82857	-0.82857	-0.65714	-0.82857	-0.82857	-0.82857	-0.82857	-0.82857	-0.48571	-0.82857	-0.54286	0.695725	0.6
Butanoic acid, hexyl ester	**	FADVs	0.542857	0.485714	0.6	0.714286	0.542857	-0.6	-0.6	-0.65714	-0.6	-0.6	-0.6	-0.6	-0.6	-0.6	-0.6	-0.48571	0.811679	0.657143
α-Terpineol	*	VTs	0.485714	-0.14286	0.314286	0.085714	0.485714	-0.6	-0.6	-0.42857	-0.6	-0.6	-0.6	-0.6	-0.6	-0.31429	-0.6	-0.31429	0.231908	0.485714
Decanal	**	others	0.657143	0.371429	0.6	0.657143	0.657143	-0.77143	-0.77143	-0.71429	-0.77143	-0.77143	-0.77143	-0.77143	-0.77143	-0.6	-0.77143	-0.54286	0.927634	0.714286
(E,E)-2,4-Nonadienal	**	others	0.657143	0.371429	0.6	0.657143	0.657143	-0.77143	-0.77143	-0.71429	-0.77143	-0.77143	-0.77143	-0.77143	-0.77143	-0.6	-0.77143	-0.54286	0.927634	0.714286
Linalyl formate	**	VTs	0.6	0.085714	0.485714	0.314286	0.6	-0.65714	-0.65714	-0.54286	-0.65714	-0.65714	-0.65714	-0.65714	-0.65714	-0.48571	-0.65714	-0.37143	0.376851	0.657143
Geraniol	*	VTs	0.485714	-0.14286	0.314286	0.085714	0.485714	-0.6	-0.6	-0.42857	-0.6	-0.6	-0.6	-0.6	-0.6	-0.31429	-0.6	-0.31429	0.231908	0.485714
(Z)-2-Decenal	**	others	0.714286	0.257143	0.6	0.542857	0.714286	-0.77143	-0.77143	-0.65714	-0.77143	-0.77143	-0.77143	-0.77143	-0.77143	-0.6	-0.77143	-0.42857	0.724714	0.828571
Indole	**	AADVs	0.771429	0.371429	0.657143	0.6	0.771429	-0.71429	-0.71429	-0.6	-0.71429	-0.71429	-0.71429	-0.71429	-0.71429	-0.65714	-0.71429	-0.31429	0.666737	0.942857
Methyl geranate	**	others	0.6	0.085714	0.428571	0.314286	0.6	-0.54286	-0.54286	-0.37143	-0.54286	-0.54286	-0.54286	-0.54286	-0.54286	-0.42857	-0.54286	-0.08571	0.40584	0.828571
α-Cubebene	**	VTs	0.257143	-0.25714	0.085714	0.028571	0.257143	-0.31429	-0.31429	-0.14286	-0.31429	-0.31429	-0.31429	-0.31429	-0.31429	-0.08571	-0.31429	0.085714	0.289886	0.542857
(E)-2-Undecenal	**	others	0.657143	0.371429	0.6	0.657143	0.657143	-0.77143	-0.77143	-0.71429	-0.77143	-0.77143	-0.77143	-0.77143	-0.77143	-0.6	-0.77143	-0.54286	0.927634	0.714286
(Z)-3-Hexenyl hexanoate	**	FADVs	0.542857	-0.02857	0.371429	0.257143	0.542857	-0.6	-0.6	-0.42857	-0.6	-0.6	-0.6	-0.6	-0.6	-0.37143	-0.6	-0.2	0.463817	0.714286
(Z)-3-Hexenyl (Z)-3-hexenoate	**	FADVs	0.771429	0.542857	0.714286	0.771429	0.771429	-0.82857	-0.82857	-0.77143	-0.82857	-0.82857	-0.82857	-0.82857	-0.82857	-0.71429	-0.82857	-0.6	0.985611	0.771429
(Z)-Jasmone	**	FADVs	0.6	0.6	0.657143	0.771429	0.6	-0.54286	-0.54286	-0.6	-0.54286	-0.54286	-0.54286	-0.54286	-0.54286	-0.65714	-0.54286	-0.37143	0.753702	0.771429
β-Caryophyllen	*	VTs	0.142857	-0.2	0.028571	0.085714	0.142857	-0.2	-0.2	-0.08571	-0.2	-0.2	-0.2	-0.2	-0.2	-0.02857	-0.2	0.142857	0.376851	0.485714
α-Ionone	*	CDVs	0.085714	0.142857	0.142857	0.371429	0.085714	-0.25714	-0.25714	-0.31429	-0.25714	-0.25714	-0.25714	-0.25714	-0.25714	-0.14286	-0.25714	-0.25714	0.695725	0.2
γ-Elemene	**	VTs	0.6	0.6	0.714286	0.771429	0.6	-0.65714	-0.65714	-0.77143	-0.65714	-0.65714	-0.65714	-0.65714	-0.65714	-0.71429	-0.65714	-0.65714	0.724714	0.6
(Z)-Geranylacetone	**	CDVs	0.771429	0.542857	0.714286	0.771429	0.771429	-0.82857	-0.82857	-0.77143	-0.82857	-0.82857	-0.82857	-0.82857	-0.82857	-0.71429	-0.82857	-0.6	0.985611	0.771429
Humulene	**	VTs	0.657143	0.371429	0.6	0.657143	0.657143	-0.77143	-0.77143	-0.71429	-0.77143	-0.77143	-0.77143	-0.77143	-0.77143	-0.6	-0.77143	-0.54286	0.927634	0.714286
(E)-β-Famesene	**	VTs	0.6	0.314286	0.542857	0.6	0.6	-0.65714	-0.65714	-0.6	-0.65714	-0.65714	-0.65714	-0.65714	-0.65714	-0.54286	-0.65714	-0.37143	0.811679	0.771429
(E)-2-Dodecenal	**	others	0.371429	0.371429	0.428571	0.6	0.371429	-0.54286	-0.54286	-0.6	-0.54286	-0.54286	-0.54286	-0.54286	-0.54286	-0.42857	-0.54286	-0.54286	0.869657	0.371429
β-Ionone	**	CDVs	0.6	0.542857	0.657143	0.771429	0.6	-0.71429	-0.71429	-0.77143	-0.71429	-0.71429	-0.71429	-0.71429	-0.71429	-0.65714	-0.71429	-0.65714	0.927634	0.6
(Z,E)-α-Farnesene	**	VTs	0.657143	0.371429	0.6	0.657143	0.657143	-0.77143	-0.77143	-0.71429	-0.77143	-0.77143	-0.77143	-0.77143	-0.77143	-0.6	-0.77143	-0.54286	0.927634	0.714286

α-Muurolene	**	VTs	0.314286	0.085714	0.257143	0.371429	0.314286	-0.37143	-0.37143	-0.31429	-0.37143	-0.37143	-0.37143	-0.37143	-0.37143	-0.25714	-0.37143	-0.08571	0.637748	0.6
α-Farnesene	**	VTs	0.485714	-0.2	0.257143	0.085714	0.485714	-0.65714	-0.65714	-0.42857	-0.65714	-0.65714	-0.65714	-0.65714	-0.65714	-0.25714	-0.65714	-0.31429	0.434828	0.485714
δ-Cadinene	*	VTs	0.314286	0.085714	0.257143	0.371429	0.314286	-0.37143	-0.37143	-0.31429	-0.37143	-0.37143	-0.37143	-0.37143	-0.37143	-0.25714	-0.37143	-0.08571	0.637748	0.6
(E)-Nerolidol	*	VTs	0.485714	-0.14286	0.314286	0.085714	0.485714	-0.6	-0.6	-0.42857	-0.6	-0.6	-0.6	-0.6	-0.6	-0.31429	-0.6	-0.31429	0.231908	0.485714
(Z)-3-Hexenyl benzoate	**	AADVs	0.6	0.085714	0.428571	0.314286	0.6	-0.54286	-0.54286	-0.37143	-0.54286	-0.54286	-0.54286	-0.54286	-0.54286	-0.42857	-0.54286	-0.08571	0.40584	0.828571
Geranyl isovalerate	**	AADVs	0.657143	0.371429	0.6	0.657143	0.657143	-0.77143	-0.77143	-0.71429	-0.77143	-0.77143	-0.77143	-0.77143	-0.77143	-0.6	-0.77143	-0.54286	0.927634	0.714286
T-Muurolol	**	VTs	0.6	0.314286	0.542857	0.6	0.6	-0.65714	-0.65714	-0.6	-0.65714	-0.65714	-0.65714	-0.65714	-0.65714	-0.54286	-0.65714	-0.37143	0.811679	0.771429
α-Cadinol	**	VTs	0.314286	0.085714	0.257143	0.371429	0.314286	-0.37143	-0.37143	-0.31429	-0.37143	-0.37143	-0.37143	-0.37143	-0.37143	-0.25714	-0.37143	-0.08571	0.637748	0.6
Expert			0.753702	0.637748	0.753702	0.840668	0.753702	-0.81168	-0.81168	-0.81168	-0.81168	-0.81168	-0.81168	-0.81168	-0.81168	-0.7537	-0.81168	-0.66674	1	0.724714
Consumer			0.885714	0.542857	0.771429	0.714286	0.885714	-0.77143	-0.77143	-0.65714	-0.77143	-0.77143	-0.77143	-0.77143	-0.77143	-0.77143	-0.77143	-0.37143	0.724714	1

Notes: Expert： the average score of expert sensory evaluation. Consumer: the average score of consumer acceptance evaluation
